# Warming Houses

**Published:** 1874-12

**Authors:** 


### WARMING HOUSES.

To warm a house healthfully there must be a constant change of air—pure air coming in, foul air going out. This is what is meant by a draught or ventilation. If no weather-strips are used, a large amount of pure out-door air will come in at the joinings of the doors and windows ; and if there is an open fire-place, or an ordinary grate with fire, the out-door air, being heavier and stronger than that in-doors, drives it up the chimney—displaces it. Hence, weather-strips are health-destroyers, because they prevent the incoming of out-door air—prevent the ventilation of an apartment.

A few years ago an English family, of parents and six children, all occupying a single room, were, one by one, taken ill. Medical skill was unavailing, and several of the members died. Being winter time a very small fire was kept up, but every crevice in the cottage was carefully stuffed with such materials as might be found in such a household. The physician in attendance had frequently directed an airing of the apartment, but the inclemency of the weather, and the danger of draughts upon the sick, was constantly interposed as an objection. All medicine seemed to fail of its ordinary good effects, and the sick failed to improve. At this juncture a window glass was accidentally broken out, and there being in that out-of-the-way poor place no ready means of repair the "air-hole" remained unaltered, resulting in an immediate improvement of the sick, and final restoration. No death occurred after the breaking of the window-pane. Within a few years a yellow fever hospital was set on fire near New York, and burnt down, the patients having been all removed previously, and distributed about on their beds on the grass. The weather was inclement at the time, and it was considered a most inhuman proceeding ; but all the sick recovered. These, and other facts like them, show the value of out-door air in curing the sick ; and if it does that, it may well be supposed to be a more efficient agent in preventing diseases.

There is only one good, healthy method of warming a family room in a northern winter, and that is, a broad, open fire-place, with wood as fuel—but wood is too expensive for general use—and next to that is bituminous or soft coal. It burns with a cheerful flame like wood, and creates a constant draught up the chimney ; but soft coal is too expensive for the masses, and hard coal must supply its place. But the ordinary grate is too small in its dimensions ; and, being nearly a foot above the level of the floor, the feet, which rest on the floor and need to be kept comfortably warm, more than any other part of the body, must suffer from getting uncomfortably cold all the time. Besides this, a common grate is nothing more than a square hole in the wall, and thus so little heat is thrown out into the room that one-third of it is wasted, being driven up the chimney, the more so as the surface of the fire is so small that, where the coals are all aglow, the apartment is not completely warm, even on a moderately cold day. The remedy for this is a broad fire-place, on a level with the floor, with pleasing jaws and a large throat—simply an old-fashioned fire-place. By an ingenious expedient, Dixon and Sons, of Philadelphia, have contrived to make the hardest coal kindle and burn, without the use of a blower, in just such a fire-place. It is called "The Low-down Grate," and is one of the greatest comforts in modern housekeeping, and is in very common use in Philadelphia.

